# Role of Surface Energy of Nanoparticle Stabilizers in the Synthesis of Microspheres via Pickering Emulsion Polymerization

**DOI:** 10.3390/nano12060995

**Published:** 2022-03-17

**Authors:** Andrei Honciuc, Oana-Iuliana Negru

**Affiliations:** Electroactive Polymers and Plasmochemistry Laboratory, “Petru Poni” Institute of Macromolecular Chemistry, Aleea Gr. Ghica Voda 41A, 700487 Iasi, Romania; negru.oana@icmpp.ro

**Keywords:** pickering emulsions, colloidosomes, polymer microspheres, nanostructured surfaces, surface energy, wettability of nanoparticles

## Abstract

Polymer microspheres are important for a variety of applications, such as ion exchange chromatography, catalyst supports, absorbents, etc. Synthesis of large microspheres can be challenging, because they cannot be obtained easily via classic emulsion polymerization, but rather by more complex methods. Here, we present a facile method for obtaining polymer microspheres, beyond 50 μm, via Pickering emulsion polymerization. The method consists in creating oil-in-water (*o*/*w*) Pickering emulsion/suspension from vinyl bearing monomers, immiscible with water, whereas silica nanoparticles (NPs), bearing glycidyl functionalities, have a stabilizing role by adsorbing at the monomer/water interface of emulsion droplets. The emulsion is polymerized under UV light, and polymer microspheres decorated with NPs are obtained. We discovered that the contact angle of the NPs with the polymer microsphere is the key parameter for tuning the size and the quality of the obtained microspheres. The contact angle depends on the NPs’ interfacial energy and its polar and dispersive contributions, which we determine with a newly developed NanoTraPPED method. By varying the NPs’ surface functionality, we demonstrate that when their interfacial energy with water decreases, their energy of adhesion to water increases, causing the curvature of the polymer/water interface to decrease, resulting in increasingly larger polymer microspheres.

## 1. Introduction

A plethora of technologies and materials continuously emerge from Pickering emulsions, which can be oil-in-water (*o*/*w*), water-in-oil (*w*/*o*), or oil-in-oil (*o*/*o*), between immiscible mineral and silicon oils, stabilized solely by particles. Recent reports [1,2] give a comprehensive overview on the type and application of Pickering emulsions. Some emerging technologies based on Pickering emulsions are edible coatings and antioxidant coatings for the food industry [3,4], emulsion liquid membranes [5], manufacturing of porous materials for wastewater treatment [6], photocatalysis [7], creams and lotions for topical drug delivery [8,9], etc. Virtually all types of oils, ranging from natural oils, water immiscible oils, essential oils, monomers, silicon oils, etc., can be emulsified with the appropriate particle stabilizers, which can be polymeric, such as well-defined polystyrene nanoparticles [10,11], Janus nanoparticles [12,13], chitosan [14], or inorganic, silica nanoparticles [15,16], laponite clay [17], hydroxyapatite [18], magnetic Fe_3_O_4_ nanoparticles [19], carbon nanotubes [7], or natural stabilizers, such as soy and whey proteins, starch, zein [20], bacteria [21], and nanocellulose [22]-related particles. Pickering emulsions can be biocompatible if the oils and stabilizing particles are biocompatible such as starch [9]. The emulsion droplet protected by an armor of self-assembled monolayers of nanoparticles, referred to as colloidosomes [14], are attractive vehicles for microencapsulation and cascade reactions [15], or even as platforms for fundamental studies for interfacial dynamics such as diffusion, interaction, 2D assembly of particles at liquid–liquid interfaces [23,24,25,26], etc. The application potential of Pickering emulsions technology in manufacturing nano and microstructures can be greatly expanded by combining it with complementary methods; for example, micron sized hollow spheres decorated with catalyst nanoparticles could be obtained [27] from combining Pickering emulsification with non-solvent induced phase separation (NIPS) method. NIPS is a widely used method for fabrication of polymeric membranes and microspheres [28,29,30]. Another particularly attractive aspect related to Pickering emulsions is the possibility to polymerize them, when the oil phase is a water immiscible vinyl bearing monomer; this way a plethora of new polymeric nano- and micromaterials can be obtained, including molecular imprinted polymers [6,22,31], colloidal particles with structural complexity [32], conductive nanostructured microcapsules [33], organic-inorganic composite particles [34], etc. In a recent report, we have shown that the Pickering emulsion polymerization technology can be used to measure the surface energy and surface energy components of the emulsion stabilizing particles [35].

How are the Pickering emulsions stabilized by particles? In essence, the problem has been already addressed since 1923, at least at the conceptual level [36], when Finkle et al. [37] suggested that the particles used as stabilizer of emulsions have to be wetted to some degree by both phases, the oil and water, in order to adsorb at the oil–water interface. Furthermore, the difference in wettability “bends” the interface in the direction of more poorly wetting liquid, thus causing its emulsification and breaking into droplets. Thus, if it were possible to measure the wettability of particles by water and oil, one can predict the type of emulsion generated *o*/*w* or *w*/*o* based on the difference in wettability. In 2001, Binks and Clint [38], and in a later work by Aveyard [39], tried to predict the Pickering emulsion phases from the relative interaction strength of the NP-oil and NP-water as reflected in the magnitude of interfacial energies between the three phases of an emulsion. They have calculated the contact angle of a theoretical NP with various oils and water, starting with literature data of the surface energy and its polar and disperse components. They arrived at the conclusion that for a pristine silica NPs whose contact angle with the water remains <75° and with the oil > 105° can only stabilize *o*/*w* emulsions. Vice versa for hydrophobically modified silica NPs, whose contact angle with water is always >130° and with oil < 50°, can only stabilize *w*/*o* emulsions. It is also implied in these works that an emulsion phase inversion to take place, from *w*/*o* to *o*/*w* and vice versa, must pass through a contact angle of 90°. Thus far, to the best of our knowledge there has not been a quantitative experimental confirmation for these theoretical predictions, because measuring the contact angle of the particles has been an ongoing challenge for fundamental science [35,40,41]. Further, there are no current studies linking the contact angle with the emulsification ability of NPs in terms of size of the emulsion droplets generates, nor the curvature of the oil/water interface upon adsorption of NPs at the interface. In this work, we address exactly this aspect and demonstrate experimentally that indeed the above theoretical predictions are correct and further, we provide a tested method to measure the contact angle of NPs and their interfacial energy via Pickering emulsion polymerization. The novelty of the current work is on one hand to provide experimental proof of the relationship that exists between the surface properties of the stabilizing NPs and the size of the emulsion droplet that can be obtained. On the other hand, we show that based on these parameters, the Pickering emulsion polymerization is a facile route toward obtaining large polymer microspheres with nanostructured surface. Further, we emphasize the determinant role of interfacial energy and its components of the NPs in determining the size of polymer microspheres that can be obtained by Pickering emulsion polymerization and thus provide a route to tuning the size of spherical microparticles with nanostructured surface.

## 2. Materials and Methods

### 2.1. Materials

Tetraethylorthosilicate (TEOS) 99%, (3-glycidoxypropyl)trimethoxysilane (GLYMO) 98% and benzoin methyl ether (BME) 97% were purchased from abcr GmbH, Karlsruhe, Germany. Divinylbenzene (DVB) technical grade—80%, benzyl methacrylate (BM) 96% containing monomethyl ether hydroquinone as an inhibitor, 2-(Dimethylamino)ethyl methacrylate (DAEMA) contains 700–1000 ppm monomethyl ether hydroquinone as inhibitor 98%, tert-butyl acrylate (tBA) 98% containing 10–20 ppm monomethyl ether hydroquinone as an inhibitor, methyl methacrylate (MM) 99% stabilized for synthesis with monomethyl ether hydroquinone as an inhibitor, ammonium hydroxide solution (28–30%) for analysis EMSURE ACS. Reag. Ph Eur. Supelco and aluminum oxide (active basic) Brockmann I, were purchased from Sigma-Aldrich (Merck KGaA, Darmstadt, Germany). Ethanol absolute (EtOH, 99.3%) and hydrochloric acid (HCl) were purchased from Chemical Company S.A. Iasi, Romania.

### 2.2. Synthesis of Silica Nanoparticles

In a 1000 mL round-bottom flask, 9 mL TEOS, 300 mL EtOH, 33 mL H_2_O, and 27.7 mL NH_4_OH were added. The reaction mixture was stirred at room temperature at 1000 rpm. A second mixture of 54 mL TEOS and 200 mL EtOH was added drop-wise via separatory funnel for about 3 h. The reaction mixture was left for 24 h at room temperature. At the end, the reaction mixture was neutralized with 18 mL of HCl. After reaction, the NPs were separated by centrifugation and washed three times with EtOH and three times with water. For the washing cycles of the NPs, ultasonication was used to redisperse the particles in the desired solvents, and centrifugation was used in every step of solid–liquid separation. At the end, the NPs were dispersed and kept in EtOH.

### 2.3. Surface Modification of Silica NPs with (3-Glycidoxypropyl)trimethoxysilane (NPs-Gly)

Pristine silica NPs were functionalized with (3-glycidoxypropyl)trimethoxysilane to obtain silica NPs bearing glycidyl surface functionality (NPs-Gly), according to Scheme S1. For this purpose, 6.0 g of silica nanoparticles (40 mL of EtOH) were added in a 250 mL flask. Then, 100 mL EtOH was added, and the mixture was kept under nitrogen atmosphere. The reaction mixture was stirred at 1000 rpm. Subsequently, 10 mL of (3-glycidoxypropyl)trimethoxysilane was added drop by drop. At the end of the addition time, the reaction mixture was heated and maintained at 60 °C for 24 h. The functionalized NPs were washed three times with EtOH and another three times with water, and were finally redispersed in water. SEM analysis indicated that the obtained silica NPs size was approximately 560 ± 3 nm, which had the tendency to pack and self-assemble (Figure S1). Zeta potential (ζ) analysis was done before and after the surface modification, Table S1. The samples were prepared with the same concentration for a better comparation. The comparative Fourier transform infrared (FTIR) spectra of silica NPs and NP-Gly are shown in Figure S2 and demonstrate successful functionalization; see spectra analysis and discussion in the Supplementary Information (SI).

### 2.4. Pickering Emulsion Preparation and Polymerization

In this experiment, the solid NPs participate in the emulsification of the monomers in water. The chemical structure of the monomers used for Pickering emulsification and polymerization are given in Figure S3. The emulsion droplets stabilized by the NPs are often referred to as “colloidosomes”. The inhibitors present in the monomers were removed by passing them through basic alumina columns. The concentration of NPs, ratio of the monomer to water, and the sonication amplitude was kept constant throughout this work, see the summary of the emulsification receipts in Table S2. For example, in a 20 mL vial were added 20 mg BME initiator, then 1 mL monomer and 0.1 mL DVB crosslinker, next, the mixture were left to rest for 3 min to produce a homogeneous solution. Finally, 5 mg colloidal particles and 12 mL water was added. Then, the glass scintillator vial was sonicated, while cooling the vial in ice and acetone bath, for 15, 30, and 45 s at 30% amplitude with a Branson 450 Sonifier equipped with a 7 mm diameter horn, which corresponds to an effective energy input of ≈217, 435, and 652 J, respectively. The acoustic energy input was and its effect on the emulsion was discussed in detail in a previous report from our group [42]. Subsequently, the prepared Pickering emulsions were placed under the UV lamp (wavelength = 365 nm, with 4 lamps, each with an intensity = 2.2 mW/cm^2^) for 1 h to initiate the polymerization reaction. At the end of the reaction time, the samples were sonicated in an ultrasonic bath for 1 min, then the polymerization product was filtered and washed with 30 mL EtOH to remove the unreacted monomer and left to dry at room temperature.

### 2.5. Measurement of the Contact Angles for NP-Gly

The measurement of the contact angle for NP-Gly was done according to the recently reported NanoTraPPED method [35]. The interfacial immersion depth of the nanoparticles trapped at the polymer-water interface during the Pickering emulsion formation and polymerization were studied with a Verios G4 UC Scanning Electron Microscope (SEM) from Thermo Fischer Scientific (Eindhoven, The Netherlands) in field immersion mode, using either a Through Lens Detector (TLD) or Mirror Detector (MD), using a stage bias of 1000 mV, a beam energy between 500 eV and 1 KeV, and an aperture of 500 pA-1 nA, to minimize the surface charging effects and also the impact of the electron beam on polymers. The contact angle was determined from the circular traces left by NP-Gly on five polymer surfaces: PMM, PEM, PBM, PDAEMA, and PtBA, see Figure S3.

## 3. Results

### 3.1. Pickering Emulsion Formation and Polymerization

We have investigated the formation and polymerization of Pickering emulsions from three different monomers: methyl methacrylate (MM), benzyl methacrylate (BM), and tert-butyl acrylate (tBA), with three types of silica NPs bearing three different surface functional groups: nitrile (NP-CN), glycidyl (NP-Gly), thiol (NP-SH). In all cases, we kept the same concentration of NPs and the same water to oil ratio in emulsion preparation, Table S2. The preparation procedure for the NP-CN and NP-SH was reported previously [35]. The synthesis of NP-Gly is given in the experimental methods and SEM images are given in the Figure S1.

**Figure 1:**
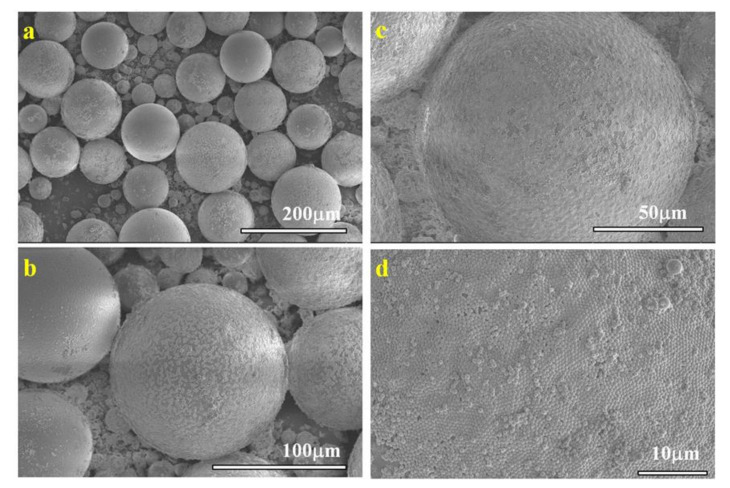
SEM images of PMM microspheres at different magnifications: (**a**) 200×, (**b**) 500×, (**c**) 800×, and (**d**) 2500×, resulted from the polymerization of Pickering emulsions that varies in size and curvature depending on the surface energy of the nanoparticles. In (**d**), the hexagonal close-packed lattice of the self-assembled monolayer of NPs can be seen.

The obtained emulsions, containing the oil soluble benzoin methyl ether (BME) initiator, were polymerized under exposure to UV lamp for 1 h, as described in the experimental section. Poly(methyl methacrylate) (PMM), poly(benzyl methacrylate) (PBM), and poly(tert-butyl acrylate) (PtBA) microspheres were obtained for each monomer and each particle, except for NP-SH with MM and NP-CN with MM, for which Pickering emulsions could be generated, but no polymer microspheres could be obtained. Qualitatively, from the SEM images, we observe that the microspheres obtained are large and uniform, especially for those obtained by NP-Gly with PMM (Figure 1), PBM (Figure S4), and PtBA (Figure S5), while for the other NPs, the microspheres appear somewhat smaller, their size distribution is broader and deviate somewhat from ideal spherical shape, see Figure S6.

In all cases, the microspheres obtained are covered by a self-assembled monolayer of NPs, see Figure 1d, which suggests that the initiation of the polymerization took place in the liquid emulsion droplet. Sometimes smaller microspheres can be identified without NPs on their surface, Figure S7. This can be explained by the polymerization mechanism of Pickering emulsions, which is rather different from the polymerization mechanism of the classic emulsions stabilized by surfactants. On one hand, the mechanism in conventional emulsion polymerization stabilized by surfactants is well-understood, whereas the standard theory notes the existence of three different *loci* for the nucleation mechanism, micellar nucleation, homogeneous coagulative nucleation, and emulsion droplet nucleation [43,44]. Although, almost always, the three mechanism are at play in the emulsion polymerization, the dominant mechanism depends on the emulsion type, such that the polymerization *loci* can shift out of the emulsion droplet toward micelles or the water phase driven by many factors, such as emulsion droplet curvature (emulsion droplet curvature 1/R, R is the radius, increases in the order: emulsions, miniemulsions, microemulsions), by the oil–water interfacial tension, solubility of the monomer in water, the solubility of the polymerization initiator in water or oil, etc. [43] On the other hand, in the case of Pickering emulsions, in the absence of surfactants, the micellar nucleation is excluded, therefore Dai et al. [34] proposed that only two possible mechanisms are present in the initial stage of Pickering emulsion polymerization, namely (i) the homogeneous coagulative nucleation, and (ii) nucleation in the emulsion droplet. In the first case, the oligomers produced in the water phase coagulate to form nuclei that are subsequently swollen by incoming of the monomers from the emulsion droplet reservoir. Emulsion stabilizing nanoparticles attach onto these nuclei and stabilize them, and these ultimately grow into sub-micron particles. An indication that this mechanism is at play is that the obtained polymer particles are only loosely covered with nanoparticles. In contrast, in the second case, when the polymerization is initiated into the emulsion droplet, these transform into solid particles without significant size growth. This mechanism should generate rather large micron size polymer particles. We believe that an indication that this mechanism is at play is that the surface of these particles is covered by a compact monolayer of NPs, which adsorbed and self-assembled at the interface during emulsion formation prior to polymerization. This hypothesis stood at the base of developing a new method, called NanoTraPPED, for measuring the contact angle and surface energy of nanoparticles [35].

The clear mechanistic picture in Pickering emulsion polymerization is further complicated by other factors, such as the water solubility of the monomers, which can drive the migration of the nucleation *loci* from the emulsion droplet into the water phase. Some other studies [45] link the mechanism of nucleation to the solubility of the initiator, for example, when the initiator is soluble in the water phase such as ammonium persulfate (APS) homogeneous nucleation mechanism, probably around the nanoparticle stabilizers present in the water phase, resulting in polymer particles that are loosely covered with nanoparticle stabilizers. We believe this is also the case for NP-SH with MM and NP-CN with MM, for which Pickering emulsions could be generated, but no polymer microspheres could be obtained, suggesting that the polymerization *loci* are not in the monomer oil colloidosome, but have shifted into the water phase. This way, nano and microparticles have formed from the nuclei in the water phase, consuming the monomers in the colloidosomes, and can be easily identified in the SEM images, because they bear no NPs, Figures S6 and S7. The NPs used initially in stabilizing the colloidosomes in the emulsion formation adsorb due to mechanical energy input, but do not spontaneously adsorb at the newly formed polymer particle surfaces. The same hypothesis was supported by Bon et al. [46], who consider that the emulsion stabilizing particles do not adsorb at the interface of the nucleating particles, but that their surface wettability is initially modified by the adsorption of the oligomers and thereafter these are capable of stabilizing the growing polymer particles. Thus, when measuring the diameters of the microspheres, we did not consider those that were not covered by a monolayer of NPs, i.e., those formed by the competing mechanism of water nucleation.

### 3.2. Determining the Interfacial and Surface Energy of NPs

**Figure 2:**
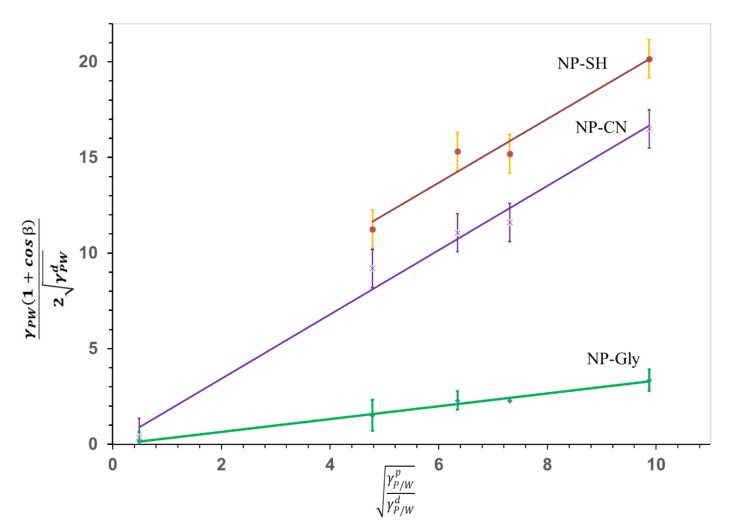
The graphical representation of the linearized OWRK Equation (1), constructed for NP-SH, NP-CN, and NP-Gly. The slope $\sqrt{\gamma_{NP/W}^{p}}$ is proportional to the polar component of the surface energy of the NPs and the intercept $\sqrt{\gamma_{NP/W}^{d}}$ is proportional to the dispersive component of the surface energy of the NPs. The data corresponding to NP-SH were offset by +3, for better visualization. The data for NP-SH, NP-CN were taken from reference [35].

The interfacial energy of the NP-CN and NP-SH with its polar $\gamma_{NP/W}^{p}$ and dispersive $\gamma_{NP/W}^{d}$ components was previously determined using the newly developed NanoTraPPED method by our group [35]. The interfacial energy with its polar and dispersive components of NP-Gly was determined in this work, using NanoTraPPED and the Owens–Wendt–Rabel–Kaelble (OWRK) [47,48] model given by the following equation:

(1) $$\frac{\gamma_{P/W}\left(1+\cos\beta \right)}{2\sqrt{\gamma_{P/W}^{d}}}=\sqrt{\gamma_{NP/W}^{p}}\sqrt{\frac{\gamma_{P/W}^{p}}{\gamma_{P/W}^{d}}}+\sqrt{\gamma_{NP/W}^{d}}$$

where, *β* is the contact angle of the NPs at the three-phase line: NP-polymer (NP/P), NP-water (NP/W), polymer-water (P/W), as indicated by the subscript to the Greek letter γ throughout this work, $\gamma_{P/W}^{p}$ and $\gamma_{P/W}^{d}$ are the polar and dispersive polymer/water interfacial energy, $\gamma_{NP/W}^{p}$ and $\gamma_{NP/W}^{d}$ are the polar and dispersive nanoparticle/water interfacial tension and $\gamma_{P/W}$ is the interfacial energy of polymer/water. Equation (1) was solved graphically, see Figure 2, by building a system of equations relating the contact angle *β* of the NP-Gly, on five different polymers listed in Table S3, with the surface energy components $\gamma_{NP/W}^{p}$ and $\gamma_{NP/W}^{d}$. Whereas the values for the polymer, $\gamma_{P/W}^{p}$ and $\gamma_{P/W}^{d}$ are known [35]. The contact angle was measured from the diameter of the circular voids or traces left be the NP-Gly on the surface of the polymer microspheres resulted from the Pickering emulsion polymerization and are shown in Figure S8, together with the obtained microspheres. The calculated contact angles are related to the diameter of the circular traces of the voids through the following geometric relationship: $\beta =\pi -\sin^{-1}\frac{l}{r}$. The total surface energy and its components $\gamma_{NP/W}^{p}$, $\gamma_{NP/W}^{d}$ in water, are obtained by fitting the data to the linear Equation (1), where the $\sqrt{\gamma_{NP/W}^{p}}$ is the slope and the $\sqrt{\gamma_{NP/W}^{d}}$ is the intercept, as shown in the Figure 2. With this, the actual values of the *γ^d^_NP/water_*, *γ^p^_NP/water_*, and *γ_NP/water_* are calculated and are given together with those for NP-CN and NP-SH in Table 1 and in Figure 2.

The total NP/w interfacial energy *γ_NP/water_*, Table 1, decreases in the order NP-SH > NP-CN > NP-Gly, suggesting the NP-Gly are wetted the most by water and are the most hydrophilic, while the NP-SH are the least wetted by water. Here we note that a vanishingly small value of the interfacial energy, see Table 1, implies complete wetting of a solid surface by a liquid and high work of adhesion energy, such as it is the case for NP-Gly, *γ_NP/water_* = 0.11 mJ/m^2^. Where, the relationship between the interfacial energy and adhesion energy is given by:

(2) $$\gamma_{NP/water}=\gamma_{NP}+\gamma_{water}-W_{NP/water}^{adhesion}$$

where the work of adhesion is:

(3) $$W_{NP/water}^{adhesion}=2\sqrt{\gamma_{NP}^{p}\gamma_{water}^{p}}+2\sqrt{\gamma_{NP}^{d}\gamma_{water}^{d}}$$

However, a vanishingly small interfacial energy of the dispersive component *γ^d^_NP/water_* ≈ 0 mJ/m^2^, such as it is the case for NP-Gly and NP-CN, indicates that the dispersive adhesion energy per unit area (adhesion forces per unit length) between the solid NP and water is comparable with the cohesion energies arising due to the dispersive interactions, in the two phases [43].

Further, the NP/w interfacial energies *γ_NP/water_*, $\gamma_{NP/W}^{d}$, $\gamma_{NP/W}^{p}$ in Table 1, are converted into surface energy of nanoparticles in air, NP/air, $\gamma_{NP}^{d}$, $\gamma_{NP}^{p}$, using the combining rules [35,43] formula:

(4) $$\gamma_{NP}^{p}={\left(\sqrt{\gamma_{NP/W}^{p}}-\sqrt{\gamma_{NP/W}^{p}}\right)}^{2}$$

The obtained values are listed in Table 2. In this case, the total surface energy *γ_NP_* is the highest for NP-Gly and decreases in the order NP-Gly > NP-CN > NP-SH. Further, while the value of the dispersive surface energy component is comparable for all three NPs, the polar surface energy component is the highest for NP-Gly and decreases in the order NP-Gly > NP-CN ≈ NP-SH, suggesting that indeed the NP-Gly are the most polar NPs, probably due to a proper functionalization but also due to orientation of the dipole moment of the glycidyl functionality (dipole moments of the functional groups: -CN, 3.4D [49], -SH = 1.39 D [50], -Gly = 2.19 D [51]). These quantitative results obtained for the surface energy of NPs will be used to interpret the emulsification and emulsion polymerization results.

## 4. Discussion

### 4.1. Role of Interfaces in Pickering Emulsion Polymerization

The calculated values for the work of adhesion between the nanoparticle and water (NP/w) and nanoparticle and polymer (NP/P) based on Equation (3) are given in Table 3. From the data, for NP/w, the magnitude of the work of adhesion decreases in order NP-Gly > NP-CN > NP-SH. The work of adhesion between the nanoparticle and polymer (NP/P) is significantly smaller than for NP/w and decreases in magnitude in the same order NP-Gly > NP-CN > NP-SH for all polymers. This confirms on one hand that the NP-Gly are most wetted by water and on the other hand that all the NPs are more strongly wetted by water than by the polymer and monomer.

At this point, it is important to note that the surface energy of the monomer and its homopolymer in the cases of those that are polymerized through radical polymerization are close, the ratio of the surface tension of the monomer to the surface energy of the polymer is proportional to the square of polymer density divided by the monomer density, typically a factor of 1.1 [52],γPγM=ρPρM2. Therefore, throughout this work we note that the contact angle determined for NPs with the polymers, by measuring the diameters of the circular traces left on the surface of the microspheres are equivalent to the contact angle of the NPs with the monomer (oil) in the emulsion droplet (colloidosome), from which microspheres are generated by polymerization to the extent given by the above relationship. Further, the contact angle of NPs will determine the size of the emulsion droplet and as soon as the polymerization starts, the contact angle will only slightly change until the equilibrium value with the polymer is reached, as the monomer is consumed, and more polymer is formed. As the time scale needed for polymerization of the emulsion droplets is 60 min, there is enough time for equilibration of the contact angle of NPs. Also, from the SEM studies, we observed that a change in density during the transition of the monomer to polymer causes [53] a small waviness of the surface of the final polymer microsphere. In this case, regions in the monolayer of NPs covering the microsphere could be jammed, being slightly pushed out or into the surface, decreasing or increasing the observed contact angle. While this jamming effect broadens the error, the average of the data should still represent the true value of the contact angle of the NPs with the polymer and as already mentioned with the monomer. However, as it can be seen in the SEM images of the Figure 1d, this effect is negligibly small for the polymers we investigated but can be better observed in the Figure S7 (top left corner of the image).

The difference in the magnitude of the work of adhesion for NPs with water vs. NP with the polymer, is reflected well in the fact that these NPs generate only *o*/*w* type emulsions from which polymer microspheres can be obtained. Next, we analyze the factors affecting the size of the microspheres obtained by emulsion droplet polymerization of the Pickering emulsions.

### 4.2. Microsphere Size Function of the Pickering Emulsions Preparation Conditions

**Figure 3:**
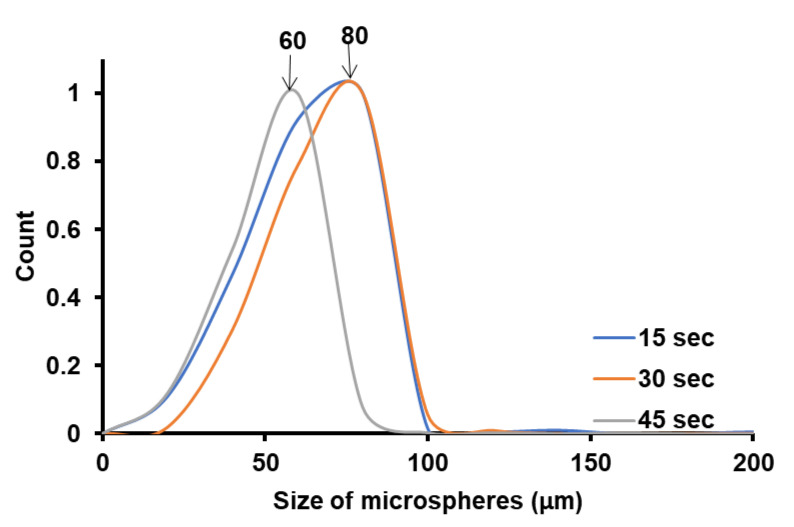
The PBM microsphere size distribution obtained by Pickering emulsion polymerization of BMand stabilized by NP-Gly at different emulsion ultrasonication times.

We have investigated the effect of ultrasonication time on the polymer microsphere size obtained from Pickering emulsion polymerization, by keeping the same intensity, the same monomer (MM), and the same nanoparticle (NP-Gly), and the data are presented in Figure 3. For the other monomers, the data are presented in SI, Figures S9 and S10.

The size distributions of the microspheres were constructed by measuring the diameters of ca. 500 microspheres from the SEM images with the ImageJ software; the SEM images were taken from different regions of the sample at a magnification of 80·. The size distribution for the microspheres obtained is rather broad, probably due to the non-uniform distribution of the acoustic field, causing different cavitation intensities in the sample volume. From Figure 3, a general trend can be seen, in the size distribution of the PBM polymer microspheres obtained. The size of the obtained PBM microspheres decreases only very slightly with the increase in the ultrasonication time, from 80 μm at 15 s to 60 μm at 45 s. This aspect is important in understanding the effect of the ultrasonication time in controlling the size of the Pickering emulsion colloidosomes and the resulting polymer microspheres. The fact that the size of the microspheres are smaller with the increase in the ultrasonication time is an expected result, and was experimentally demonstrated in surfactant-free emulsion polymerization [42]. Moreover, it can seem that the size of the polymer microspheres depends only slightly on the ultrasonication time, but as we will see next to a much lesser extent than the on the surface and interfacial energy of the NPs, that it ultimately determines the quality and size of the microspheres (colloidosomes) obtained.

### 4.3. Size of the Microspheres Function of Monomer Type for the Same NP-Gly

**Figure 4:**
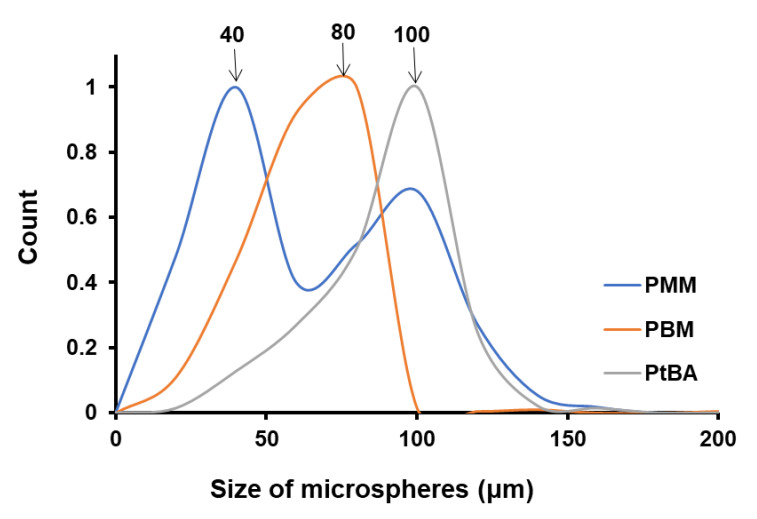
Colloidosome size distribution resulted from Pickering emulsion polymerization obtained from MM, BM, and tBA and stabilized with the NP-Gly. The ultrasonication time for the emulsion was 15 s, for all samples.

We have investigated the role of the monomer on the size of polymer microspheres obtained by Pickering emulsion polymerization, by keeping the ultrasonication time constant and utilizing the same nanoparticle (NP-Gly). In this case, the variables that change are: (i) the thickness of the monomer/water interface dictated by the solubility of the monomer in water and (ii) the immersion depth of the NP-Gly into the polymer. By plotting the microsphere size distribution of PMM, PBM, and PtBA, Figure 4, Figures S11 and S12, we find that the average microsphere size increases in the order PMM < PBM < PtBA, while we utilize the same NP-Gly in the Pickering emulsion stabilization of MM, BM, and tBA, see Figure 4. The size distribution for PMM microspheres is bimodal, one band with a maximum at ca. 40 μm and a second one with the maximum at ca. 100 μm. One possible explanation for the presence of a bimodal distribution only for PMM microspheres is that MM monomer has the highest solubility (7.4 g/L) compared to the other monomers, BM (0.4 g/L) or tBA (1.5 g/L). We hypothesize that during polymerization, the MM monomers dissolved in water could re-absorb into the polymerizing emulsion droplet and consequently generate larger microspheres. This hypothesis is supported in part by the fact that with increase in ultrasonication time, the population of the second band in the bimodal distribution increases, see Figure S9, and its maximum shifts to lower values. Because the position of the first band in the bimodal size distribution for PMM microspheres, with its maximum at 40 μm, does not shift with the ultrasonication time, we take these as the representative maximum size of the microspheres resulting from the originally formed emulsion droplets stabilized by the NPs. Further, we also observe in Table 3 that the work of adhesion of NP-Gly decreases in the order PMM > PBM > PtBA, which suggests that there is an inverse relationship between the work of adhesion of NPs to the polymer vs. the size of the obtained microspheres.

At the same time, the difference Δ*W* between the work of adhesion of NPs minus the work of adhesion of NPs to the polymer:

(5) $$\Delta W=W_{NP/water}^{adhesion}-W_{NP/P}^{adhesion}$$

increases in the order PMM < PBM < PtBA, which suggests a directly proportional relationship with the microsphere size. The calculated values for ΔW are given in Table S4.

As we already alluded above, the immersion depth of the NPs into the polymer is an expression of the affinity of the NP-Gly to the monomer and this is quantitatively expressed by the contact angle of the NPs at the three-phase line, and *β* with the monomer, which is preserved for the polymer, after the emulsion polymerization. Thus, the contact angle can be experimentally determined from the diameter of the traces left by the NPs on the colloidosome surfaces, and the found values for the NPs with the three polymers PMM, PBM, and PtBA are summarized in Table 4. From the data, the highest contact angle *β*, means that the nanoparticles are least immersed in that polymer, and it appears that they generate the largest oil/water (and after polymerization polymer/water) curvature. To summarize, the experimental data show that there is a relationship of direct proportionality between the magnitude of the contact angle *β* and the difference in the work of adhesion ΔW with the microsphere size.

### 4.4. Size of Microspheres Function of NP Type for the Same Monomer

**Figure 5:**
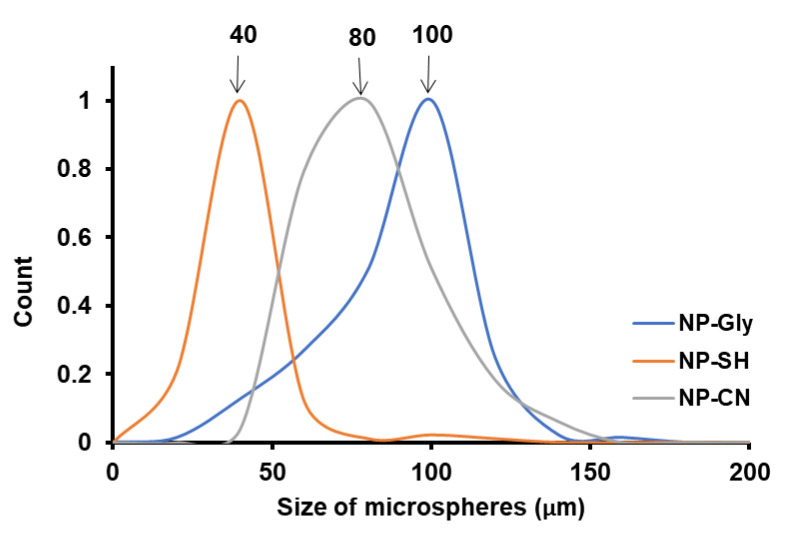
Normalized size distribution of the PtBA microspheres obtained from Pickering emulsion polymerization of tBA monomer with NPs bearing different surface functional groups NP-Gly, NP-CN, and NP-SH.

We have investigated the role of the NPs on the size of microspheres obtained by Pickering emulsion polymerization, by keeping the ultrasonication time constant and utilizing the same monomers. In this case, the only variable that changes is the immersion depth of the NP-Gly, NP-CN, and NP-SH into the polymer. By plotting the size distribution of the polymer microspheres obtained with the NP-Gly, NP-CN, and NP-SH, we find that the average microspheres size increases in the order NP-SH < NP-CN < NP-Gly, for the same tBA monomer in the Pickering emulsion generation and polymerization, see Figure 5. The contact angle *β* of the NP-Gly, NP-CN, and NP-SH with PtBA were also measured from the diameters of the traces left on the microsphere surface and are given in Table 4, and Figures S13 and S14. By analyzing the data in Table 4, a trend emerges, namely the contact angle of the NPs with PtBA increases in the order NP-SH < NP-CN < NP-Gly. This indicates that the contact angle or the immersion depth appears to be the main factor affecting the curvature of the emulsion droplet and of the resulting polymer microsphere. Based on these experimental findings, we note the critical role played by the NPs contact angle at the three-phase line. In fact, the explanation, we provide next, sheds light into the fundamentals of Pickering emulsion formation, offering direct evidence with respect to one of the main parameters determining the size of the microspheres obtained and above that determinant for the emulsion phase and curvature of the emulsion droplet and consequently of the microspheres’ size.

**Figure 6:**
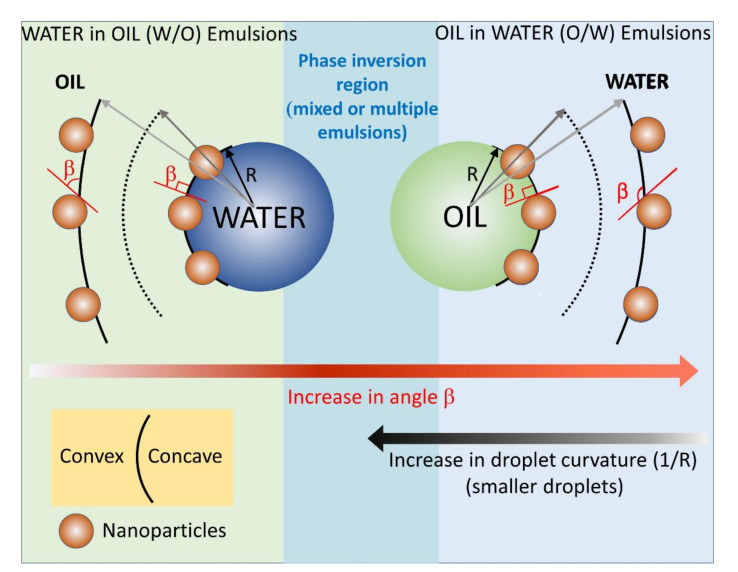
Diagram depicting the relationship between the contact angle *β* of the NP with the oil, i.e., immersion depth of the nanoparticle at the oil/water interface and the curvature of the interface. For *o*/*w* emulsions, *β* > 90°, the larger the value of the contact angle *β*, the larger the emulsion droplets. For *w*/*o* emulsions, *β* < 90°, the smaller the value of the contact angle *β*, the larger the emulsion droplets.

In the emulsion theory, such as that of Finkle and Bancroft [37,54], the emulsion type is thought to be determined by the affinity of the emulsifier surfactant or particle to either the oil or the water phase. In case of surfactants, their affinity to the oil or water phase can be gauged by a post hoc rationalization, thus a semi-quantitative criterion called hydrophilic-lipophilic balance (HLB) emerged. In the case of nanoparticles, however, such a criterion cannot be applied as it is difficult and impossible to measure an HLB value, except for Janus nanoparticles [12,55]. For homogeneous nanoparticles, we have previously hypothesized [56] that the affinity to either water or oil phases translates into a preferential immersion (or good wettability, low contact angle), of the NPs, into either one phase or the other, which ultimately will determine the curvature of the oil/water interface and the emulsion type, see the discussion in our previous publication and the cartoon in the Figure 6. The NPs’ affinity and preferential immersion of the NPs into one phase or the other can be thought of how well the NPs are wetted by one of the phases, and quantitatively by the value of the contact angle.

The cartoon in Figure 6 was constructed based on the experimental findings in Table 3, Figure 4 and Figure 5; the cartoon depicts the gradual change of the NPs immersion and contact angle *β* with the oil phase for an *o*/*w* emulsion, and its effect on the emulsion droplet radius R and curvature 1/R. Our experimental findings indicate that for an *o*/*w* emulsion, the increase in the contact angle with the oil phase, the NPs become less and less immersed in oil, the size of the obtained polymer colloidosomes also increases, e.g., the contact angle of the NPs with PtBA increases in the order NP-SH < NP-CN < NP-Gly. The value of the contact angle of the NPs with PBM also increases in the order NP-SH < NP-Gly, whereas the size of the microspheres also increases in the same order, see Figure S13. PMM only with NP-Gly microspheres could be obtained, whose size distribution is given in Figure S14. However, when the value of the contact angle *β* drops below 114° it seems that mixed or multiple emulsions are formed, as judged from the fact that no spherical microspheres are obtained anymore. Irregular polymer block shapes are obtained for NP-CN with PBM (114°), NP-CN with PMM (108°), NP-SH with PMM (106°), see Figure S15, indicating that the Pickering emulsion are complex below a critical contact angle *β*, of approximately 114° with the oil, and 66° with water, signaling the beginning of the emulsion phase inversion and the coexistence of complex emulsions, mixed and multiple emulsions. By extrapolation, we hypothesize that the same is the case for the *w*/*o* emulsions, namely that the increase in the curvature of the water emulsion droplets decreases with the decrease in the contact angle *β* of the NPs with the oil. Our experimental values for the contact angle *β* finally confirm the theoretical predictions of Binks and coworkers [38], which state that the NPs whose contact angle with the water remains <75° and with the oil > 105° can only stabilize *o*/*w* emulsions, and NPs whose contact angle with water is >130° and with oil < 50°, can only stabilize *w*/*o* emulsions, whereas in between these ranges complex multiple emulsion exist, signaling the approach of an emulsion phase inversion, when NPs have a contact angle of 90° with both phases. Further, from Table 3 and Table S4, there appears to be a relationship of direct proportionality between the increase in ΔW and the microsphere diameter.

### 4.5. Energy of Attachment of NPs at Interface vs. Size of the Microspheres

**Figure 7:**
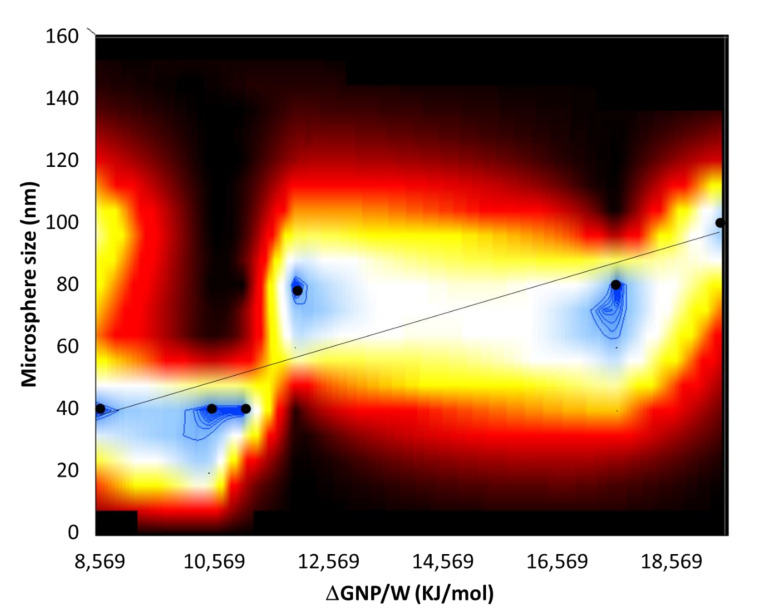
2D surface plot relating the normalized population distribution of the microspheres (*z*-axis) as a function of energy of detachment ΔG_NP/P_ of the NPs (*x*-axis) and the size of the microspheres (*y*-axis). The filled circles represent the data points corresponding to the maximum of the size distribution of the microspheres, and the black line is a guide to the eye.

Next, we attempt to understand in a different way how the curvature of the oil/water in emulsion droplet and subsequently in the resulting polymer microspheres can be influenced by the interfacial energy of the NPs and consequently by their detachment energy from water vs. oil. The free energy of adsorption and desorption of the NPs at the interfaces are related $\Delta G_{adsorption}=-\Delta G_{desorption}$. The desorption energy or the detachment energy of an NPs can be calculated from the contact angle at the interfaces and is given by the following expression [35,43]:

(6) $$-\Delta G_{detachment}=\pi R^{2}\gamma_{PW}\left(1\pm \cos\beta \right)=\Delta G_{adsorption}$$

where *γ_PW_* is the P/W interfacial energy. It is important to note that the sign in the parenthesis is taken negative when the NP detaches/desorbs from the oil/water interface into water and the sign is taken positive when the NP detaches from the oil/water interface into the oil. This distinction is important, because in the former case it gauges the affinity of the NPs with the emulsion droplets and in the latter case it gauges the affinity with the water phase. Using Equation (6) and plugging in the value for the contact angle *β*, of the NP to the polymer microspheres, calculated the detachment energies of the NPs from the polymer microspheres, ΔG_NP/P_, see Table S5, and from water, ΔG_NP/W_, see Table S6. From the values in Tables S5 and S6, the attachment energies of NPs to water are greater in magnitude than those of NPs to polymer microspheres. Further, the greatest differences in the energies of detachment of the NPs with water vs. polymer are those for the NP-Gly, followed by NP-CN and NP-SH, for each corresponding polymer. In other words, the NP-Gly has the strongest affinity toward water phase. In fact, NP-Gly also produces the largest colloidosomes, and polymer microspheres after polymerization for all type of polymers, see Figure 5 and Figure S12, also Figures S4–S6. To further emphasize this aspect, in Figure 7, we have generated a surface plot, from all NPs and monomers investigated, relating the population distribution function of the detachment energies ΔG_NP/W_ (from water) and the microsphere size. From this plot, we observe an increase in the size of the microsphere with the energy of detachment, regardless of the monomer and NP type used for generating Pickering emulsions.

These findings generate a new conjecture, namely if the curvature of the liquid menisci formed by the oil/water interface between the tightly packed NPs at the interface could determine the curvature of the entire emulsion droplet. For the case of NPs immersed less than half into the oil phase, the oil/water interfacial menisci will be convex on the water side and concave on the oil side. The NPs weakly attached to water (with small detachment energies from water), i.e., more immersed into the oil phase, will produce menisci with greater curvature than those NPs more strongly attached to water, i.e., less immersed into the oil phase, thus generating smaller emulsion droplets, with a larger curvature of the interface than the latter. This new conjecture needs a more in-depth analysis, whereas the scaling parameters must also be considered, and cannot be exhaustively treated with the available data in the current work.

## 5. Conclusions

In this work, we have presented a facile method for obtaining perfectly spherical polymer microspheres using *o*/*w* Pickering emulsions polymerization method of MM, BM, and tBA monomers, whereas three types of NPs were used as emulsion stabilizers, NP-Gly, NP-CN, and NP-SH. We have demonstrated that the key parameter influencing the size of the obtained microspheres is the surface and interfacial energy of the NPs. The surface and interfacial energy of the NPs determines their affinity toward one phase or the other and thus affects their interfacial immersion depth. Further, this immersion depth affects the curvature of the emulsion droplet and eventually of the polymer microsphere obtained after polymerization. Namely, the NPs least immersed in the emulsion droplet are NP-Gly. Further, the NP-Gly led to the formation of largest microsphere signaling that they have formed from the polymerization of a coarse Pickering emulsions, or suspensions. We have explained this by showing that NP-Gly is most strongly wetted by water, having the lowest interfacial energy with water *γ_NP/water_* = 0.11 mJ/m^2^ amongst the three NPs. This also predicts that NPs with moderate to high surface energy *γ_NP_* = 67.96 mJ/m^2^, a rather high surface polarity component *γ^p^_NP_* = 42.56 mJ/m^2^, and an extremely low interfacial energy with water, *γ_NP/water_* = 0.11 mJ/m^2^ attach poorly to oils and generate coarse Pickering emulsions, more like suspensions. From the SEM images, we observe that most perfect spheres are obtained for the most polar NP-Gly, for all three monomers investigated, completely covered by a monolayer of NPs, and we thus confirm that the polymerization *loci* reside in the suspension droplet.

On the other hand, as the surface energy of the NP-CN and NP-SH decreases, the polar contribution to surface energy also decreases, causing an overall increase of the interfacial energy with water and a deeper immersion into the emulsion droplet. These NPs generated finer Pickering emulsions and smaller microspheres after polymerization than NP-Gly. Although in the case of NP-CN and NP-SH with MM Pickering emulsions having been obtained, no microspheres were obtained after polymerization, which implies that the polymerization nucleation *locus* has completely shifted into the water phase, consuming monomer from the colloidosomes, and generating polymer nanoparticles and microparticles that are devoid of NPs on their surface. Further work should focus on fine tuning the synthesis conditions for obtaining narrower size distributions of the large microspheres from Pickering emulsions stabilized by NPs bearing polar functional groups.

## Figures and Tables

**Table 1:** Interfacial energy and its disperse $\gamma_{NP/W}^{d}$ and polar component, $\gamma_{NP/W}^{p}$ of the silica nanoparticles with water, bearing different surface functional groups. The data for NP-CN and NP-SH were taken from reference [35].

Nanoparticle	*γ^d^_NP/water_* (mN/m)	*γ^p^_NP/water_* (mN/m)	*γ_NP/water_* (mN/m)
NP-SH	0.21	3.10	3.10
NP-CN	0.00	2.77	2.77
NP-Gly	0.00	0.11	0.11

**Table 2:** Surface energy (interfacial energy of the nanoparticles with air) *γ_NP_* and its disperse $\gamma_{NP}^{d}$ and polar component, $\gamma_{NP}^{p}$ of the silica nanoparticles with water, bearing different surface functional groups. The data for NP-CN and NP-SH were taken from reference [35].

Nanoparticle	*γ^d^_NP_* (mN/m)	*γ^p^_NP_* (mN/m)	*γ_NP_* (mN/m)
NP-SH	20.96	26.60	47.56
NP-CN	25.40	26.94	52.34
NP-Gly	25.40	42.56	67.96

**Table 3:** The calculated values of the work of adhesion based on Equation (3), where the surface energy values for the NPs were taken from Table 2 and for the polymers from reference [35].

Nanoparticle	$W_{NP/water}^{adhesion}\;(\mathrm{mJ}/m^{2})$	$W_{NP/PMM}^{adhesion}\;(\mathrm{mJ}/m^{2})$	$W_{NP/PBM}^{adhesion}\;(\mathrm{mJ}/m^{2})$	$W_{NP/PtBA}^{adhesion}\;\left(\mathrm{mJ}/m^{2}\right)$
NP-SH	116.9	76.3	64.5	57.9
NP-CN	122.0	81.7	70.5	63.6
NP-Gly	140.2	87.9	72.0	64.1

**Table 4:** Summary of microsphere diameter and contact angle *β* with the polymer; the data for NP-CN and NP-SH were taken from reference [35].

	NP-SH		NP-CN		NP-Gly	
Polymer	Microsphere Diameter [μm]	*β*°	Microsphere Diameter [μm]	*β*°	Microsphere Diameter [μm]	*β*°
P(MM)	-	106.0 ± 5.2	-	108.1 ± 2.9	40, 100	149.9 ± 0.5
P(BM)	40	118.2 ± 1.9	-	114.4 ± 2.2	80	154.6 ± 0.5
P(tBA)	40	115.7 ± 2.6	80	119.4 ± 1.8	100	153.5 ± 0.9

## Data Availability

The data generated in this study is publicly available in an open access repository Open Science Framework (OSF) repository at DOI 10.17605/OSF.IO/TYM28.

## Supplementary Materials

The following are available online at https://www.mdpi.com/article/10.3390/nano12060995/s1, SEM images of the silica NPs before and after functionalization, Figure S1, Scheme S1 surface functionalization reaction scheme, Table S1, NP size and surface potentials, Figure S2, FTIR of the NPs before and after surface functionalization, chemical structure of the vinyl bearing monomers, Figure S3, recipes for Pickering emulsion preparation and polymerization Table S2, SEM images of microparticles Figures S4–S7, SEM images of the circular voids on the surface of the microparticles for contact angle measurements Figure S8, summary of the diameters and contact angles, Table S3, size distribution of the microspheres Figures S9–S14, calculated values of the work of adhesion and energies of desorption of NPs from the interface, Tables S4–S6, SEM images with the irregular shape structures vs. microspheres Figure S15.
