# Supplementary material for: Role of Surface Energy of Nanoparticle Stabilizers in the Synthesis of Microspheres via Pickering Emulsion Polymerization
Source: Nanomaterials (Basel). 2022 Mar 17;12(6):995. doi: 10.3390/nano12060995 (PMC8949673; doi:10.3390/nano12060995)
Supplement: Supplementary file 1 [file nanomaterials-12-00995-s001.zip › nanomaterials-1630894-supplementary.pdf]

Supplementary Information

Role of Surface Energy of Nanoparticle Stabilizers in the Synthesis of Microspheres via Pickering Emulsion Polymerization

Andrei Honciuc * and Oana-Iuliana Negru

Electroactive Polymers and Plasmochemistry Laboratory, "Petru Poni" Institute of Macromolecular Chemistry, Aleea Gr. Ghica Voda 41A, Iasi 700487, Romania; negru.oana@icmpp.ro

* Correspondence: honciuc.andrei@icmpp.ro

Experimental

Nanoparticle characterization

Citation: Honciuc, A.; Negru, O.-I. Role of Surface Energy of Nanoparticle Stabilizers in the Synthesis of Microspheres via Pickering Emulsion Polymerization. *Nanomaterials* **2022**, *12*, 995. https://doi.org/10.3390/nano12060995

Academic Editor: Henrich Frielinghaus

Received: 23 February 2022
Accepted: 14 March 2022
Published: 17 March 2022

Publisher's Note: MDPI stays neutral with regard to jurisdictional claims in published maps and institutional affiliations.

Copyright: © 2022 by the authors. Submitted for possible open access publication under the terms and conditions of the Creative Commons Attribution (CC BY) license (https://creativecommons.org/licenses/by/4.0/).

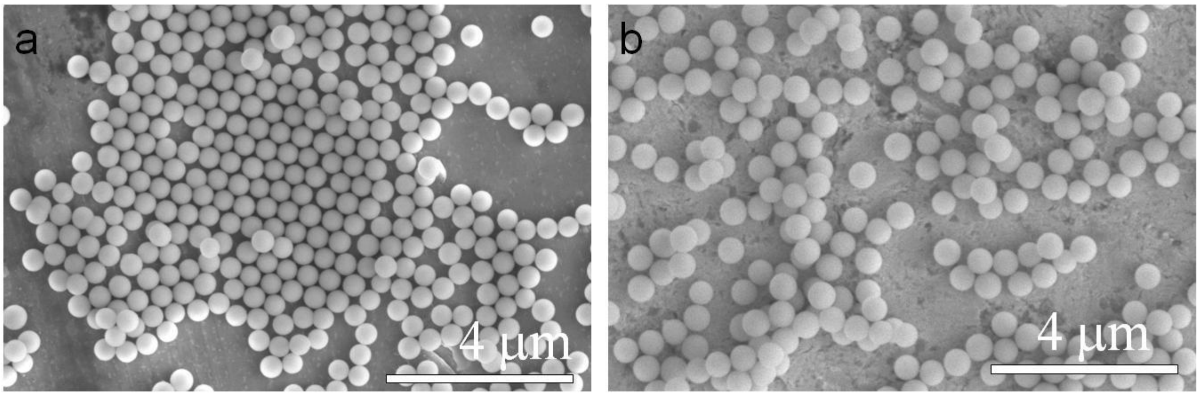

Figure S1. SEM images of silica nanoparticles **a**) before and **b**) after modification with (3-glycidoxypropyl)trimethoxysilane.

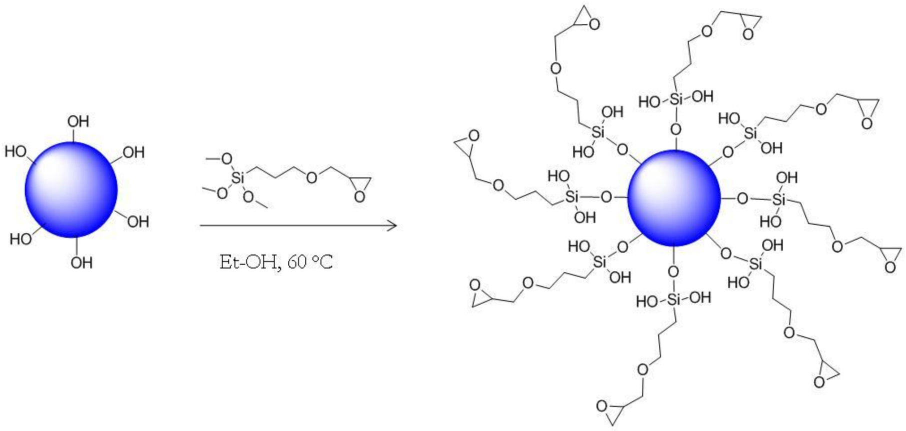

Scheme S1. Scheme of surface modification reaction of silica nanoparticles.

Table S1. Unmodified and the modified the silica nanoparticles were measured with SEM and Zeta Sizer to ζ-potential.

Functional group at the surface	Diameter / SEM [nm]	ζ-potential [mV]
NP-OH (starting material NPs)	555 ± 4	-48.3 ± 0.2
NP-Gly	560 ± 3	-44.2 ± 0.2

Figures S2 shows the FTIR spectrums of silica NPs and modified NP-Gly. Unmodified silica nanoparticles showed strong signals at 1060 cm^{-1} and 796 cm^{-1} associated with the asymmetric and symmetric stretching of Si–O–Si, respectively. The wide band at 950 cm^{-1} was due to the Si–O in plane stretching vibrations in the Si–OH group, while the peaks at 3427 and 1631 cm^{-}

[1] were attributed to the overlapped band of O–H stretching of physically adsorbed water and the Si–OH stretching of the silanol group and the O–H deformation vibration of the adsorbed water, respectively. The silica nanoparticles are composed of mainly Si–O–Si networks and include –OH and –O–CH_2–CH_3 groups as additional functional groups as well as EtOH and water as a residual component. The vibration bands at 2966 – 2852 cm^{-1} relate to the symmetric and asymmetric stretching of –CH_2 from Si–O–CH_2–CH_3.

The epoxides have characteristic IR absorption bands at 1260 – 1240 cm^{-1} (ring breathing) and 950 – 810 cm^{-1} (asymmetrical ring stretching). Another characteristic band is due to the C-H stretch in the epoxide in the region 3050 – 2995 cm^{-1}. The peaks corresponding to epoxy ring could not be clearly in the spectrum, owing to the strong presence of vibration peaks of the silica in the region. However, in the region of 800 – 1300 cm^{-1} some change take place, namely: the intensity of the band at 950 cm^{-1}decreases, and the band from 796 cm^{-1} shifting by 4 cm^{-1} and increases in intensity, this fact indicated that some silanol groups have been modifies. Furthermore, a new shoulder band at 1258 cm^{-1} can be noted.

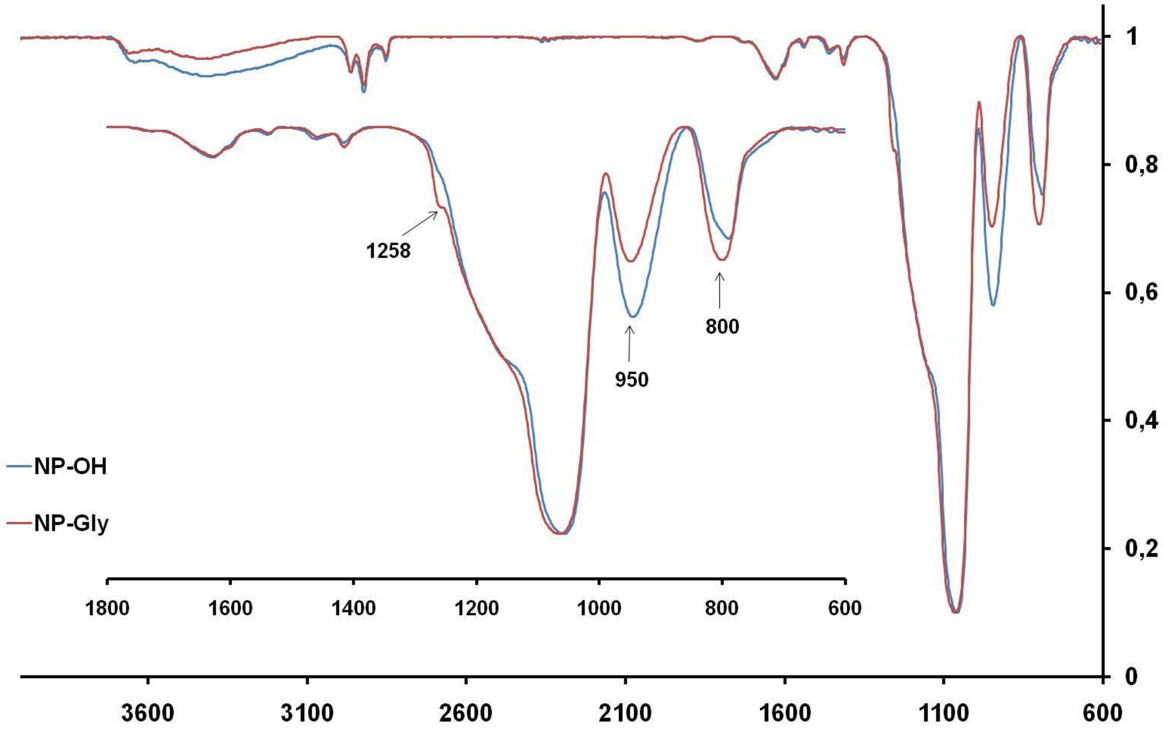

Figure S2. FTIR spectra of of silica nanoparticles before and after functionalization with (3-glycidoxypropyl)trimethoxysilane.

Pickering emulsion preparation and polymerization

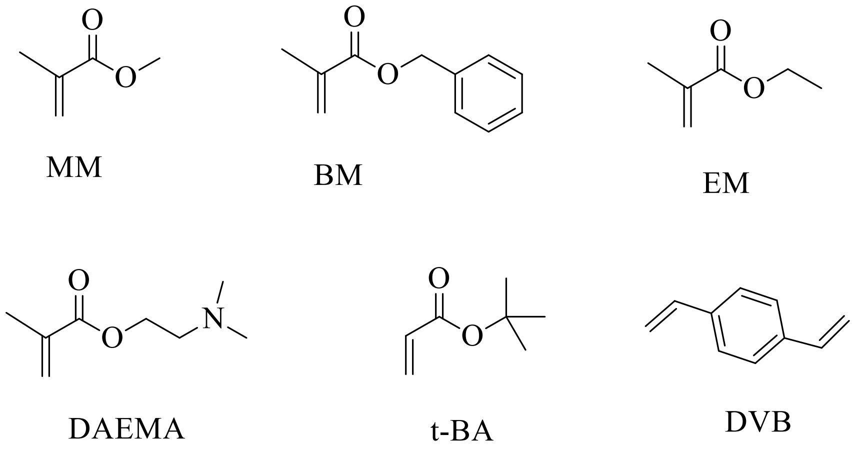

Figure S3. Vinyl bearing monomers used for the Pickering emulsion formation and polymerization.

Table S2. A summary of the conditions used in the colloidosome synthesis.

**Functional Group**	**Monomer/ [mL]**	**DVB [mL]**	**BME [mg]**	**H_2O [mL]**	**NP [mg/mL]**	**Sonication [s]**	**Amplitude [%]**
NP-Gly	MM - 1	0.1	20	12	5	15	30
	DAEMA - 1	0.1	20	12	5	15	30
	BM - 1	0.1	20	12	5	15	30
	tBA - 1	0.1	20	12	5	15	30
	EM - 1	0.1	20	12	5	15	30
	MM - 1	0.1	20	12	5	30	30
	MM - 1	0.1	20	12	5	45	30

	BM - 1	0.1	20	12	5	30	30
	BM-1	0.1	20	12	5	45	30
	tBA - 1	0.1	20	12	5	30	30
	tBA - 1	0.1	20	12	5	45	30

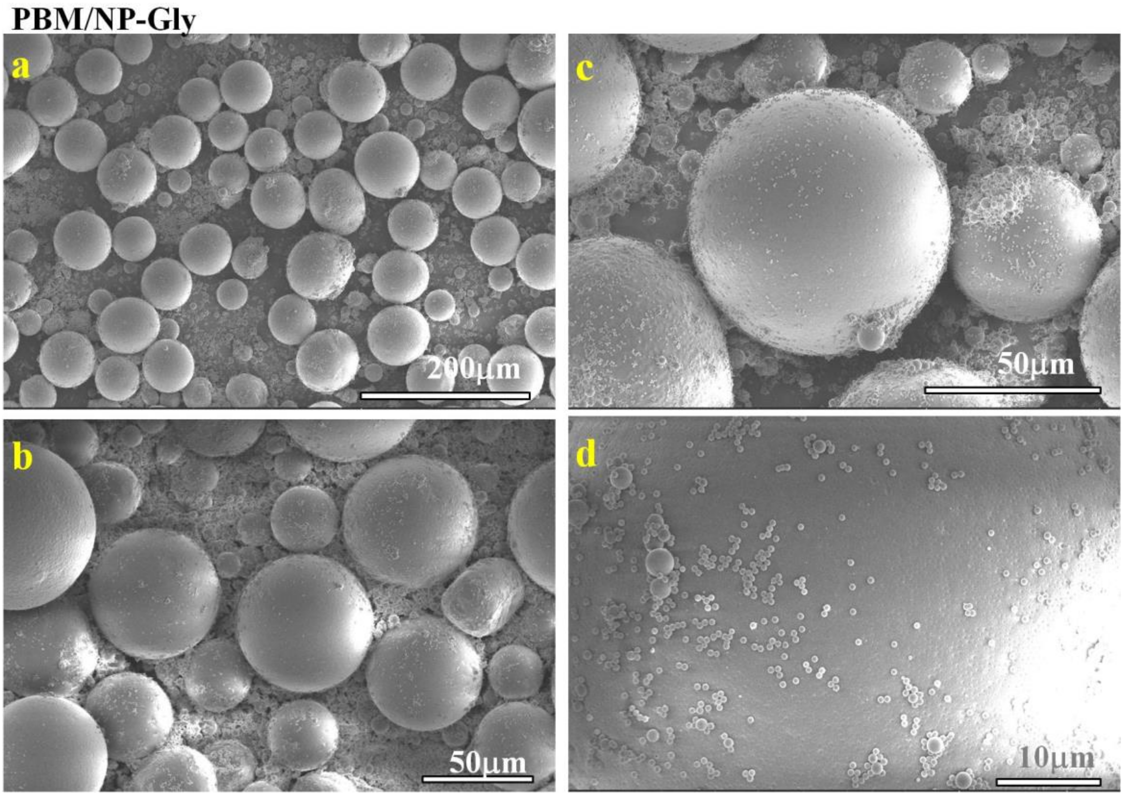

Figure S4. SEM images of PBM microspheres resulting from the polymerization of benzyl methacrylate (BM) by Pickering emulsion stabilized by NP-Gly, at four different magnifications: (**a**) 200x, (**b**) 500x, (**c**) 800x and (**d**) 2500x.

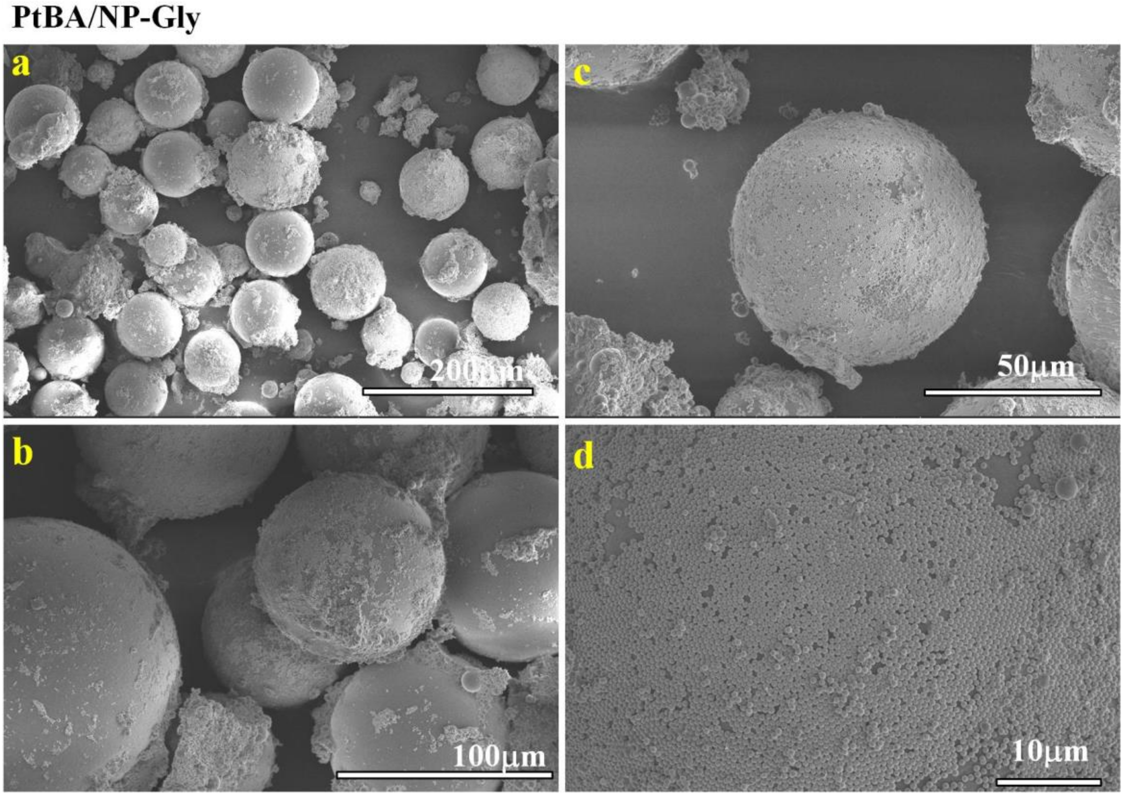

Figure S5. SEM images of PtBA microspheres resulting from the polymerization of tert-butyl acrylate (tBA) by Pickering emulsion stabilized by NP-Gly, at four different magnifications: (**a**) 200x, (**b**) 500x, (**c**) 800x and (**d**) 2500x.

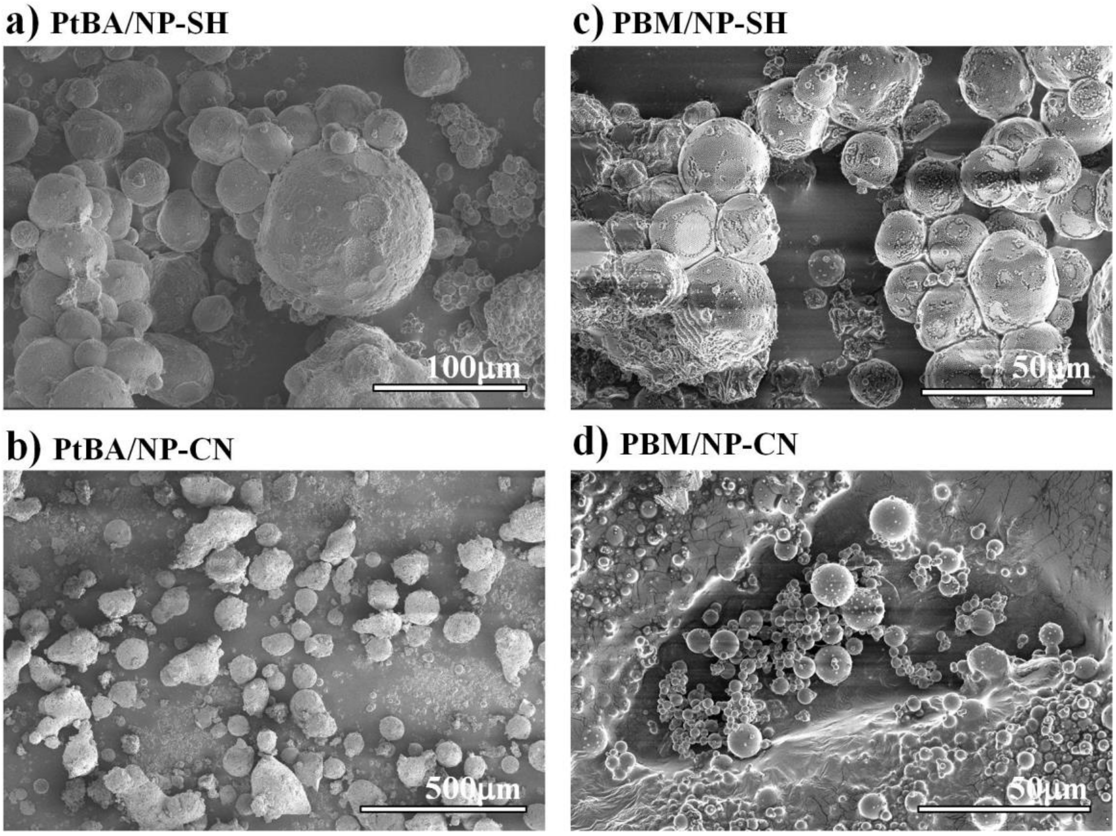

Figure S6. SEM images of microspheres resulting from the polymerization of Pickering emulsions, stabilized with NP-CN and NP-SH: (**a**) PtBA microspheres stabilized by NP-SH, (**b**) PtBA microspheres stabilized by NP-CN, (**c**) PBM microspheres stabilized by NP-SH and (**d**) PBM microspheres stabilized by NP-CN.

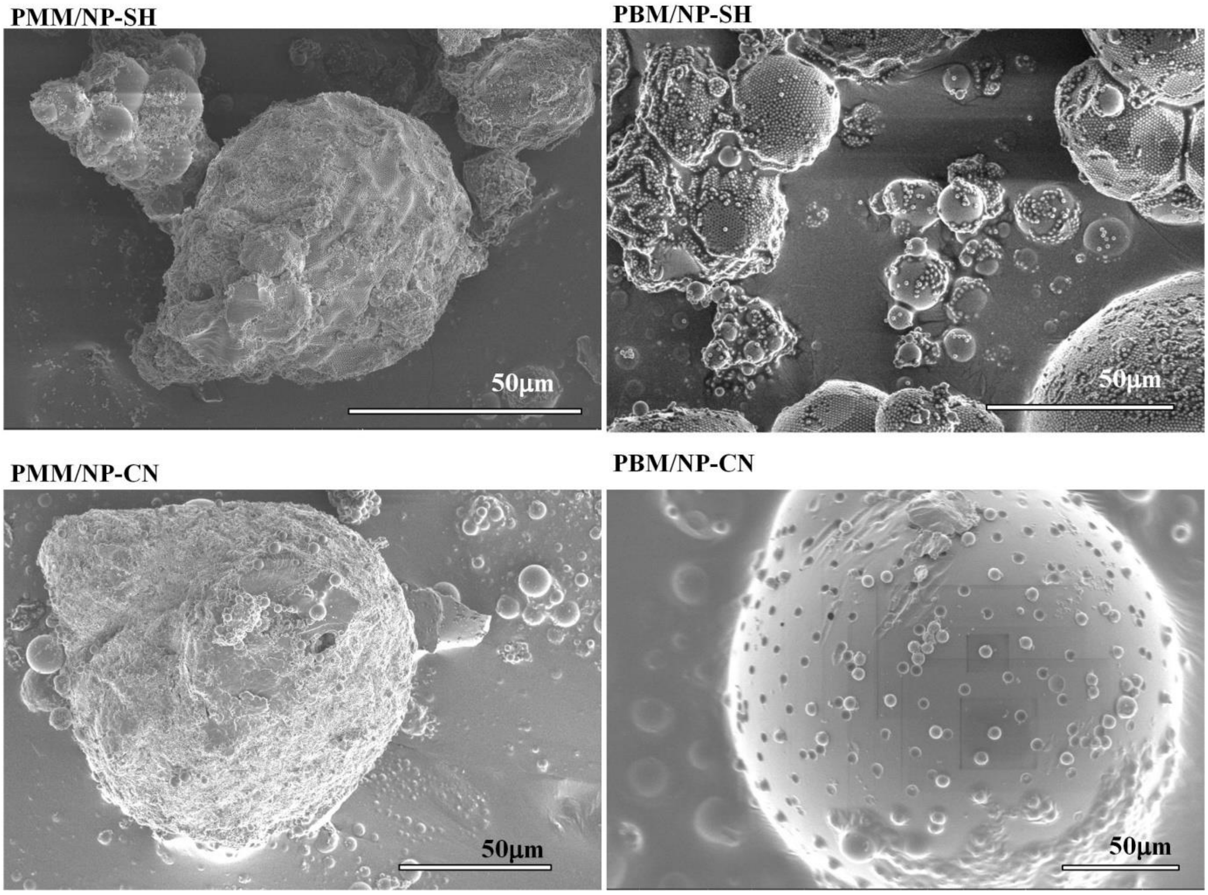

Figure S7. Various microspheres that are obtained from a concurrent polymerization mechanism, via nucleation in water, showing little or no coverage by NPs.

Contact angle measurements

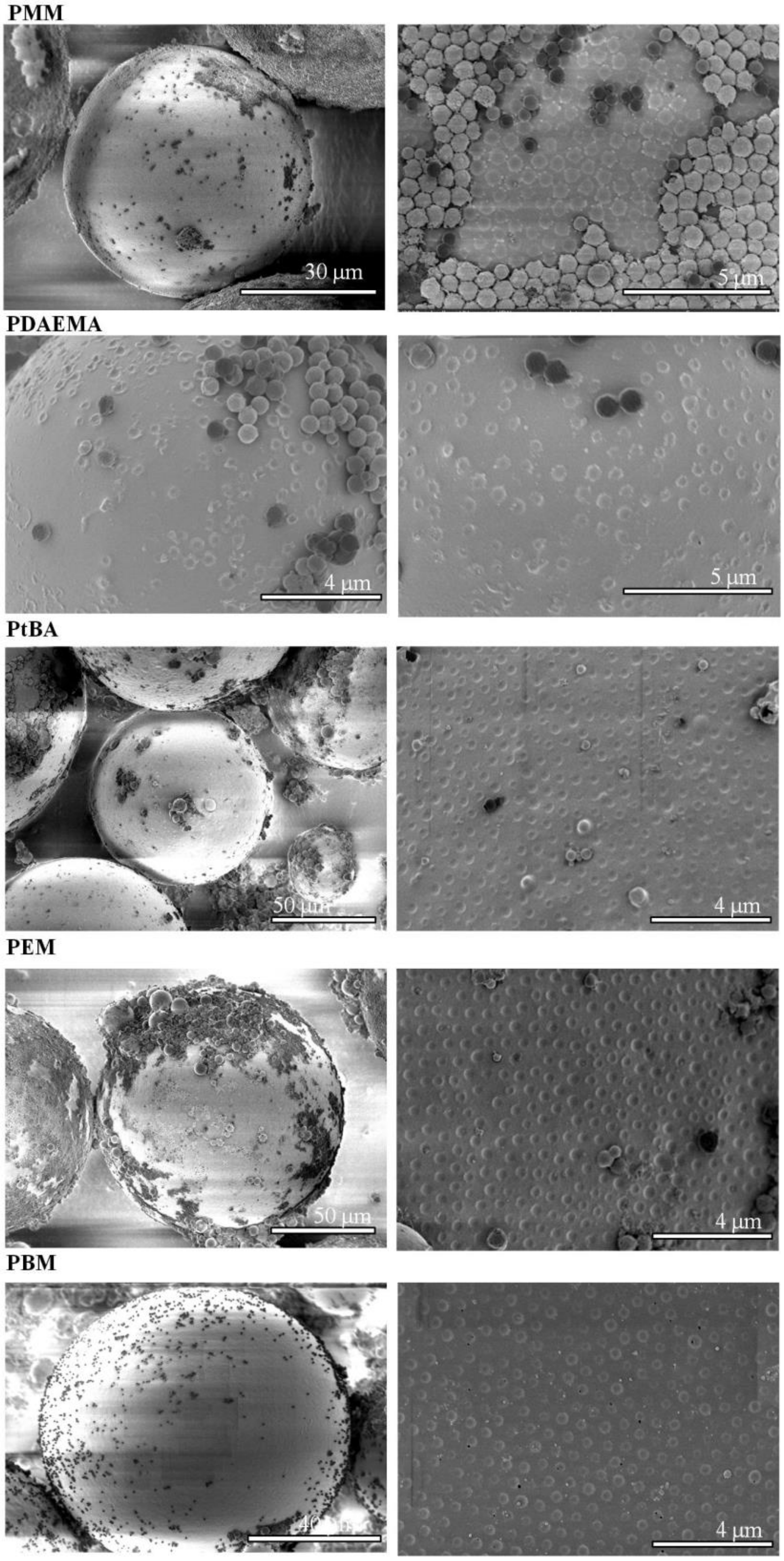

Figure S8. Left - SEM images of the polymerized o/w Pickering emulsions, consisting of spherical microparticles – colloidosomes; Right: SEM images of the surface of the microspheres showing NP-Gly nanoparticles and circular traces left after their removal by ultrasonication.

Table S3. Summary of all hole diameter, contact angle with the polymer.

Functional Group	Polymer	Hole diameter [nm]	β °
NP-Gly	P(MM)	280 ± 2.09	149.98 ± 0.49
	P(DAEMA)	300 ± 3.30	147.59 ± 0.80
	P(BM)	240 ± 2.11	154.61 ± 0.48
	P(tBA)	250 ± 1.79	153.47 ± 0.92
	P(EM)	265 ± 1.63	151.74 ± 0.38

Polymer microspheres – synthesis and characterization

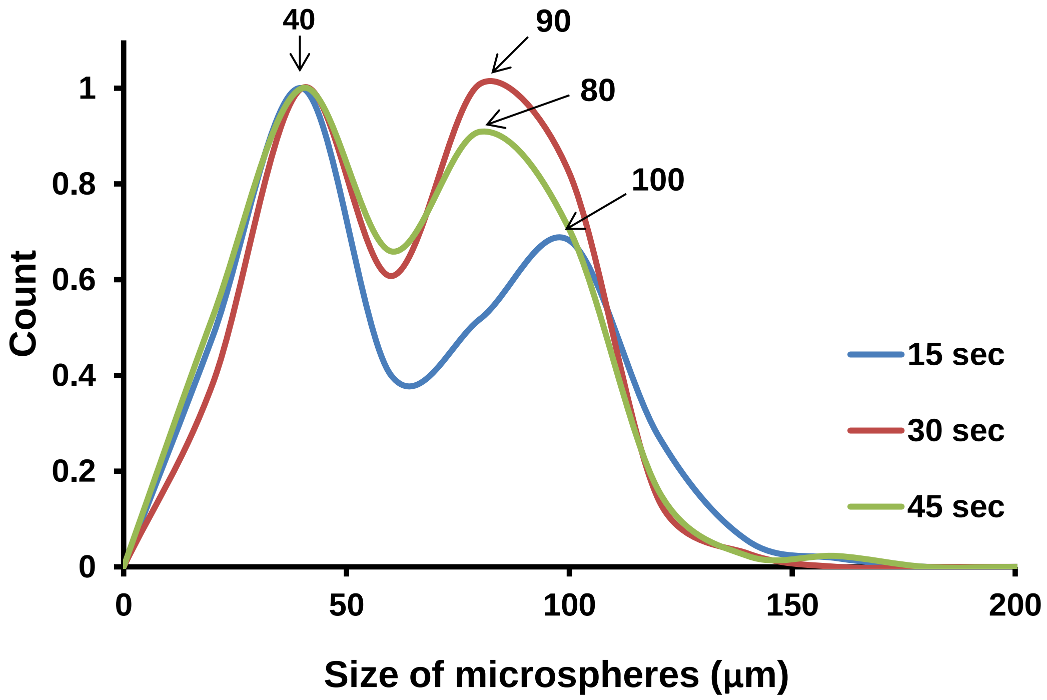

Figure S9. Normalized distribution of PMM microsphere obtained by Pickering emulsion polymerization of MM and stabilized by NP-Gly at different emulsion ultrasonication times.

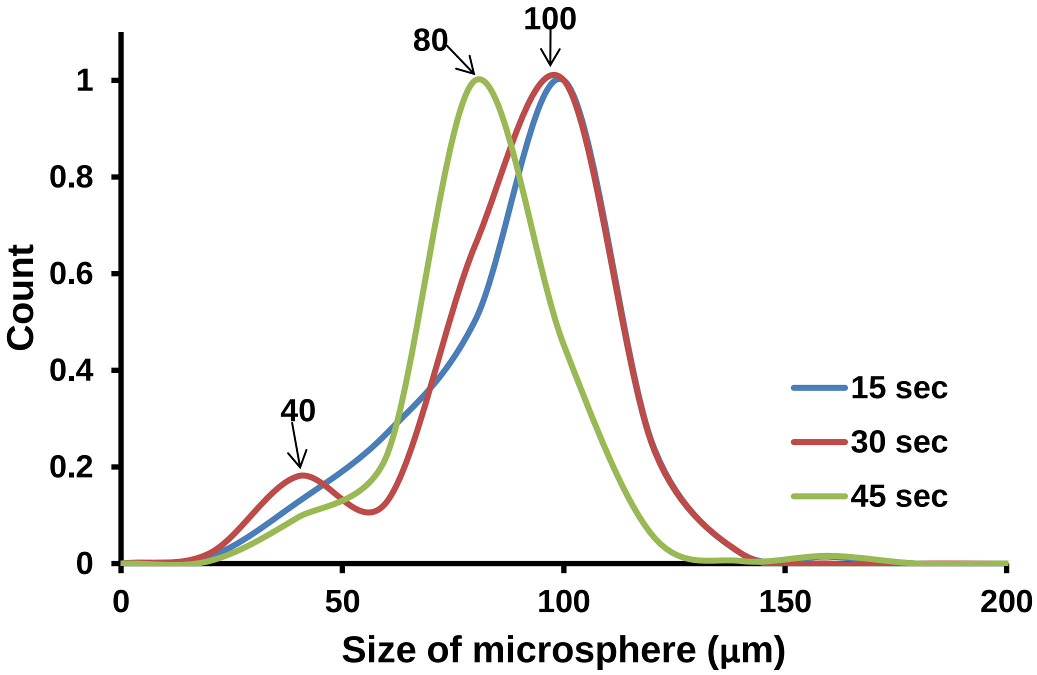

Figure S10. Normalized size distribution of PtBA microsphere obtained by Pickering emulsion polymerization of tBA and stabilized by NP-Gly at different emulsion ultrasonication times.

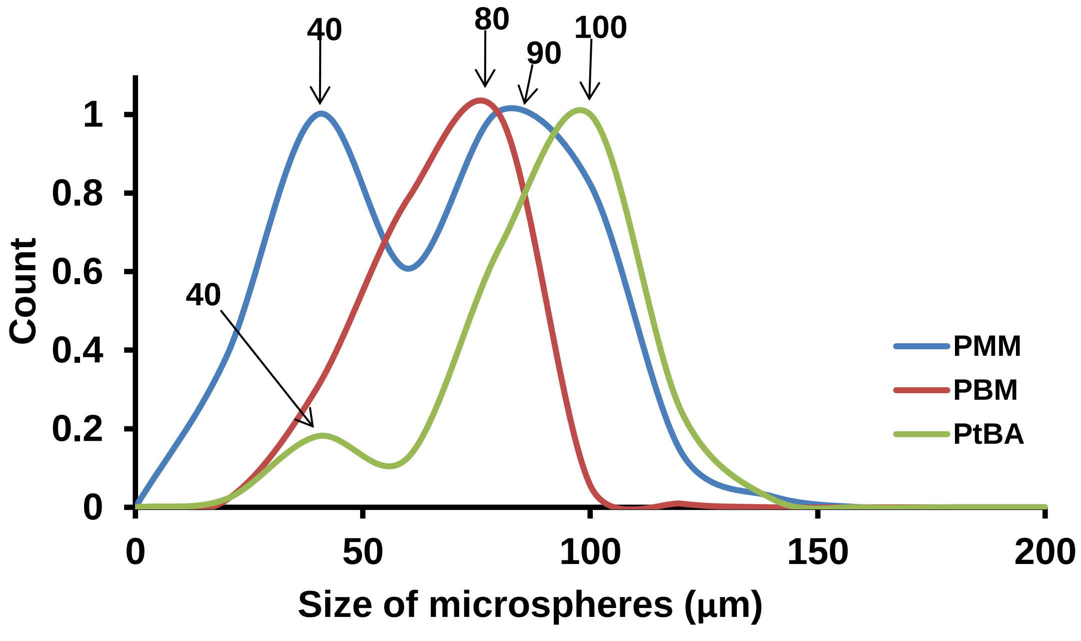

Figure S11. Normalized size distribution resulted from Pickering emulsion polymerization obtained from MM, BM and tBA and stabilized with the NP-Gly. The ultrasonication time for the emulsion was 30 s, for all samples.

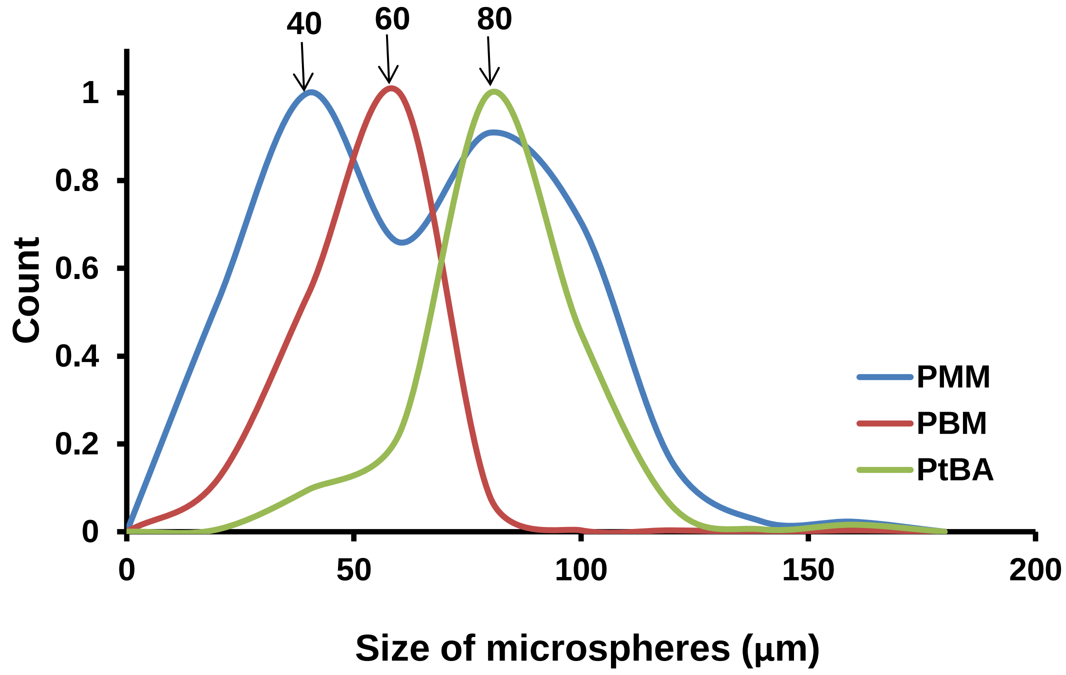

Figure S12. Normalized size distribution resulted from Pickering emulsion polymerization obtained from MM, BM and tBA and stabilized with the NP-Gly. The ultrasonication time for the emulsion was 45 s, for all samples.

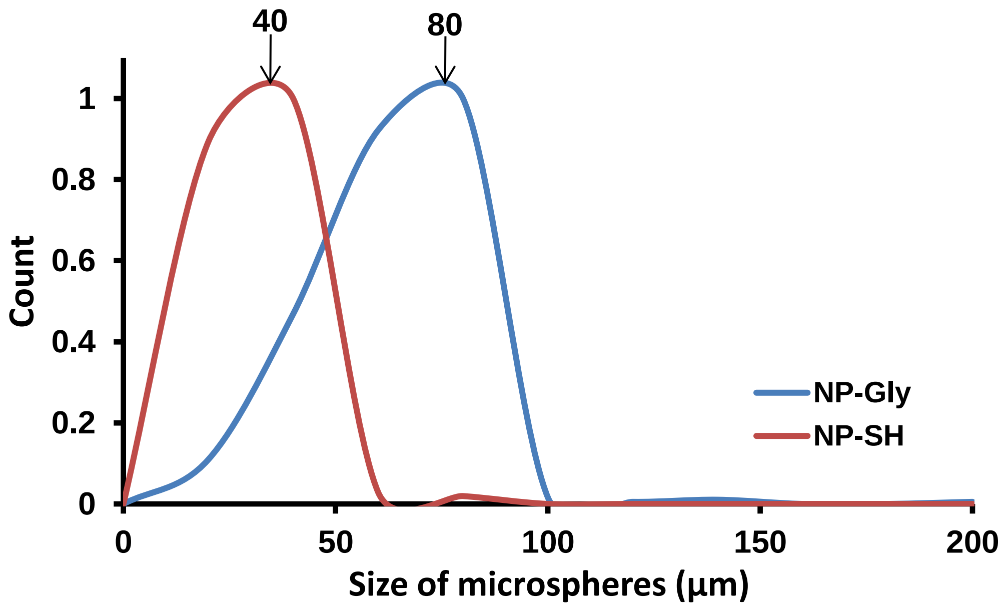

Figure S13. Normalized size distribution of the PBM microspheres obtained from Pickering emulsion polymerization of BM monomer with NPs bearing different surface functional groups NP-Gly and NP-SH.

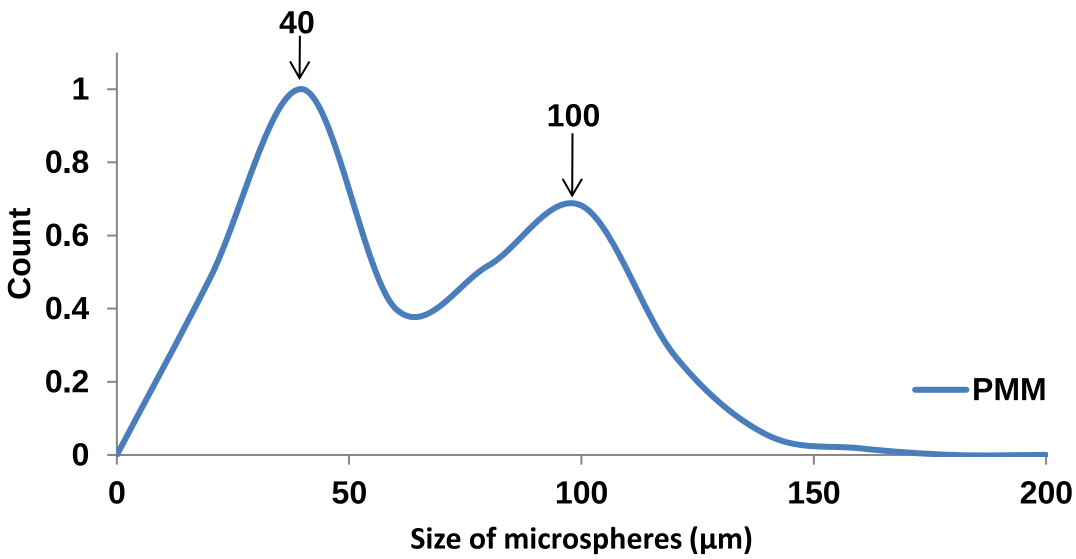

Figure S14. Normalized size distribution of the PMM microspheres obtained from Pickering emulsion polymerization of MM monomer with with the NP-Gly.

Table S4. Calculated value of the ΔW which is difference between the work of adhesion of NPs minus the work of adhesion of NPs to the polymer.

**Nanoparticle**	$W_{NP/water}^{adhesion} - W_{NP/PMM}^{adhesion}$ (mJ/m^2)	$W_{NP/water}^{adhesion} - W_{NP/PBM}^{adhesion}$ (mJ/m^2)	$W_{NP/water}^{adhesion} - W_{NP/PtBA}^{adhesion}$ (mJ/m^2)
NP-SH	40.6	52.3	58.9
NP-CN	40.3	51.5	58.4
NP-Gly	52.4	68.2	76.2

Table S5. Calculated the detachment energies of the NPs from polymer, $\Delta G_{NP/P}$.

Polymer	$\Delta G_{NP\text{-}Gly/P}$ (KJ/mol) ×10^{-3}	$\Delta G_{NP\text{-}CN/P}$ (KJ/mol) ×10^{-3}	$\Delta G_{NP\text{-}SH/P}$ (KJ/mol) ×10^{-3}
PMM	44.3	1169.4	1291.6
PBM	45.3	1674.6	1349.1
PtBA	60.0	1402.7	1736.5

Table S6. Calculated the detachment energies of the NPs from water, $\Delta G_{NP/W.}$

Polymer	$\Delta G_{NP\text{-}Gly/W}$ (KJ/mol) $\times 10^{-3}$	$\Delta G_{NP\text{-}CN/W}$ (KJ/mol) $\times 10^{-3}$	$\Delta G_{NP\text{-}SH/W}$ (KJ/mol) $\times 10^{-3}$
PMM	8569.3	4228.7	4005.1
PBM	17586.2	9686.7	10529.4
PtBA	19419.5	12022.0	11114.8

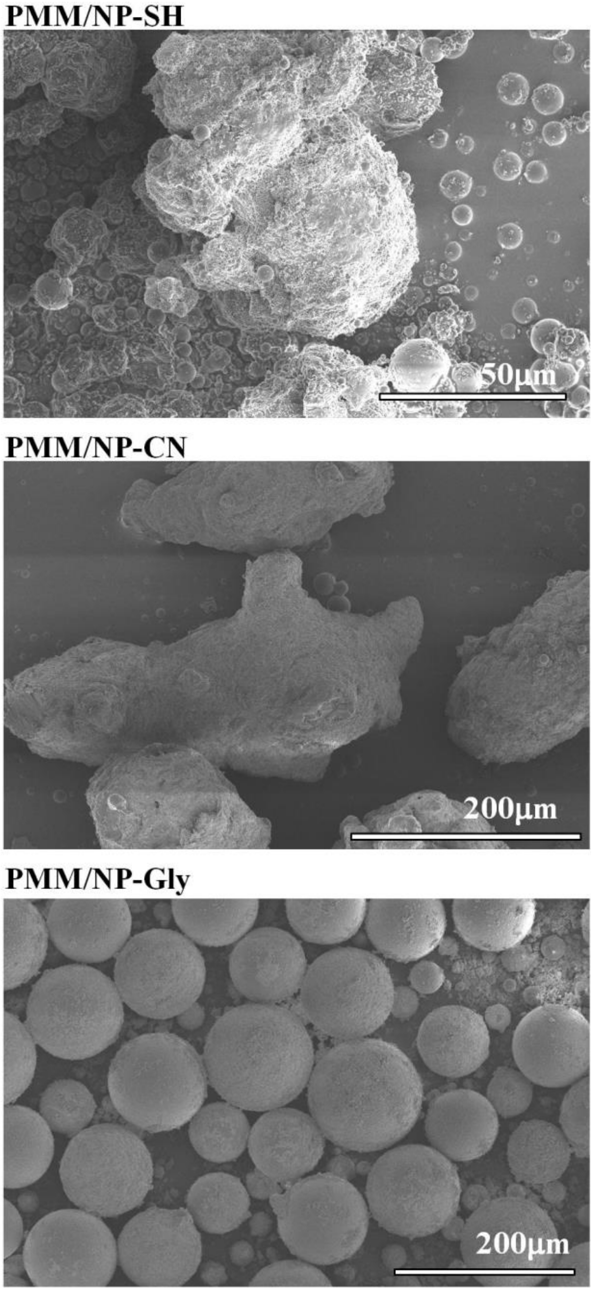

Figure S15. Irregular polymer shapes obtained from NP-SH with PMM (β=106°), NP-CN with PMM (β=108°), compared with perfectly spherical microspheres obtained from NP-Gly with PMM (β=149.9°).
